# Potential Galactagogue Effect of Tri-Than-Thip Remedy on Milk Synthesis in Lactating Rats: Impact on Milk Production-Related Parameters

**DOI:** 10.7150/ijms.83869

**Published:** 2023-07-24

**Authors:** Piriya Chonsut, Palika Wetchakul, Jomkarn Naphatthalung, Chuchard Punsawad, Fendi Pradana, Sineenart Sanpinit

**Affiliations:** 1 Department of Applied Thai Traditional Medicine, School of Medicine, Walailak University, Nakhon Si Thammarat, 80160, Thailand.; 2 Research Center in Tropical Pathobiology, Walailak University, Nakhon Si Thammarat, 80160, Thailand.; 3 Faculty of Traditional Thai Medicine, Prince of Songkla University, Songkhla, 90110, Thailand.; 4 Department of Medical Sciences, School of Medicine, Walailak University, Thasala, Nakhon Si Thammarat 80160, Thailand.; 5 Nutrition Study Program, Tadulako University, Indonesia, 94116.

**Keywords:** Tri-Than-Thip remedy, milk production, lactating rats, galactagogue, hypogalactia, Thai herbal medicine

## Abstract

Herbal galactagogues have been widely used as a treatment for postpartum hypogalactia due to the potential side effects associated with pharmacological therapy. Tri-Than-Thip (Tri-TT) is a Thai herbal medicine remedy that contains three main components: *Cassia fistula, Pithecellobium dulce, and Ficus benjamina*. These components are believed to have properties that contribute to milk production. However, despite the traditional use of Tri-TT, there is a lack of academic evidence supporting its efficacy in enhancing milk production. Therefore, the purpose of this study was to investigate the effect of Tri-TT on milk production and determine if it has a galactagogue effect. The weight suckle weight model was used to determine total milk production in lactating rats, while histological analysis was performed to assess the alveolar diameter of the mammary gland. The findings of this study revealed a significant increase in total milk production among lactating rats treated with 500 mg/kg of Tri-TT, compared to the control group. Furthermore, both the Tri-TT and Domperidone-treated groups exhibited a larger alveolar diameter of the mammary gland in comparison to the control group. In summary, these findings provide supportive evidence for the galactagogue activity of Tri-TT. The observed enhancement in milk production may be associated with Tri-TT could potentially be attributed to its ability to widen the alveolar diameter of the mammary gland, thereby facilitating increased milk volume.

## Introduction

Breast milk is a vital source of nutrition **1** that promotes cognitive and physical functions and supports the baby's healthy growth**.** It also strengthens the immune system and minimizes the risk of infection and allergies **2.** According to the World Health Organization** (**WHO**)**, newborns should be breastfed for at least six months and continued for one year or longer, which is consistent with the Thailand Multiple Indicator Cluster Survey **(**MICS**)** conducted by the National Statistical Office **(**NSO**)** in collaboration with UNICEF **3.** Nowadays, many postpartum hypogalactia women are having problems breastfeeding and their milk supply is low as a result of stress, anxiety, or maternal illness**. 4.** Metoclopramide and domperidone** (**DOM**)** are pharmacological therapies that are administered, but their use is limited due to safety concerns**. 5-7.** Herbal galactagogues are interestingly used as alternative therapies**.** The use of herbs, such as banana blossom, basil, and pumpkin has been demonstrated in various studies to increase milk production in postpartum moms **4, 8, 9.**

In Thailand, the Tri**-**Than**-**Thip **(**Tri**-**TT**)** remedy, comprising *Cassia fistula*** (**C**.** *fistula***)**, *Pithecellobium dulce*** (**P**.** *dulce***)**, and *Ficus benjamina*** (**F**.** *benjamina***)**, has been traditionally utilized for various therapeutic purposes, such as diuretic, antimicrobial, and wound healing effects**.** Additionally, it has been recognized for its potential to promote milk production **10, 11.**

Previous studies have analyzed the chemical composition of the Tri**-**TT remedy and its herbal components, revealing the presence of various bioactive compounds, such as phenolics, flavonoids, alkaloids, and glycosides **(**Table 1**)^10^.** Lin et al**.** conducted studies demonstrating that quercetin, a flavonoid found in Tri**-**TT and P**.** *dulce*, possesses milk**-**stimulating properties by stimulating prolactin, thereby aiding in milk production in mice with milk deficiency **12** In addition, (-)-gallocatechin, a compound found in F**.** *benjamina*, has been reported to have potential benefits in enhancing the quality and quantity of raw milk produced by livestock **13.** Furthermore, a study investigated the effects of condensed tannins **(**CT**)** derived from *Ficus bengalensis* leaves, as well as tannin found in *Cassia alata*, on feed utilization, milk production, and the health status of crossbred cows**.** The study revealed that supplementing crossbred cows with 1**.**5**%** CT positively influenced milk production during mid**-**lactation **14.** Overall, these findings suggest that the chemical constituents present in Tri**-**TT, including quercetin and (-)-gallocatechin, as well as tannin, may play a role in promoting milk production and improving its quality**.**

Aquaporins **(**AQPs**)** are a family of membrane proteins that play a crucial role in maintaining water balance by facilitating the movement of water across cellular membranes **15, 16.** In the context of milk synthesis and production, AQPs are involved in the transport of water and small solutes across mammary endothelial barriers **16**, While the role of AQPs in mammary epithelial cells is well**-**established, their presence and potential function in blood vessels raise intriguing possibilities**.** It is plausible that AQPs in blood vessels could participate in regulating water movement in and out of these vessels **17**, thereby potentially influencing milk production and lactation processes. In this study, we investigate the expression of AQPs in the blood**.** Specifically, focused on AQP3 and AQP5, as they have been identified as predominantly involved in milk production **5, 18, 19.**

α**-**Lactalbumin **(**LALBA**)** is a globular protein that plays a vital role in the development of infants **20.** Its involvement in milk synthesis includes its interaction with the enzyme β**-**1,4**-**galactosyltransferase, resulting in the formation of lactose synthase**.** This enzyme converts glucose and galactose into lactose, which acts as an osmotic force, drawing water into the mammary gland and contributing to increased milk production **21.** In our study, we focused on examining the impact of Tri**-**TT on LALBA levels in the blood**.** This investigation is supported by previous research conducted by McFadden, who analyzed the concentration of alpha**-**lactalbumin in the blood of cattle during different physiological states, such as gestation, the peripartum period, and lactation**.** This research suggests that monitoring changes in LALBA levels in the blood can provide valuable insights into the developmental and functional changes occurring in the mammary glands of cattle **22.** Consequently, LALBA represents an intriguing protein of interest for further investigation**.**

To examine the milk production effects of Tri**-**TT, it is important to note that there is currently a lack of evidence regarding its galactagogue activity and potential toxicity**.** Consequently, the objective of this study was to investigate the impact of Tri**-**TT on milk production in lactating rats**.**

## Materials and Methods

### Chemicals and Reagents

Aquaporin 3** (**AQP3**) (**cat no**.** SL0096Ra, Assay range**:**45 pg**/**ml **-** 4000 pg**/**ml**)**, Aquaporin 5** (**AQP5**) (**cat no**.** SL0098Ra, Assay range**:**80 pg**/**ml **-**4000 pg**/**ml**)**, Alpha**-**lactalbumin activity** (**LALBA** (**cat no**.** SL1609Ra, Assay range**:** 0**.**1 ng**/**ml **-** 10 ng**/**ml**)** kits were purchased from Sunlong Biotech Co**.**, Ltd**.**, Hangzhou, China**.** DOM used as the reference positive drug was purchased from MacroPhar Co**.**, Ltd**.**, Bangkok, Thailand**.**

### Plants Collection and Tri-Than-Thip Remedy Preparation

The medicinal plant constituents of Tri**-**TT remedy, including *P****.*** *dulce, C****.*** *fistula,* and* F****.*** *benjamina* were purchased from Triburi Orsot, a local licensed medicinal plant store in Songkhla Province, Thailand**.** Tri**-**TT was prepared by mixing 1 kg of the same ratio **(**333**.**34 g per compound**)** in distilled water and heating it for 30 minutes until it began to boil**.** the sample cools down at room temperature**.** Tri**-**TT sample was filtered using cotton and filter paper **(**Whatman No**.** 1**)** dried in a lyophilizer and stored at **-**20 °C until use**.** The weight percentage **(%** w**/**w**)** was used to determine the extraction yield of plant extract**. 10**

**Extraction yield(%)** = [(weight of dried extract)/(weight of initial dry material)] **x** 100

### Animal Preparation

Female Wistar virgin rats** (**10**-**12 weeks old**)**, weighing 200**-**250 g and pregnant Wistar rats** (**12 weeks old**)**, weighing 250**-**300 g at gestational age** (**G1**)** 14**-**16 days were purchased from Nomura Siam International Co**.**, Ltd**.**, Bangkok, Thailand**.** They were housed in separate cages at a constant temperature of 22 ±1 °C and relative humidity of 50 ± 3**%**, with a 12**-**hour light**-**dark cycle**.** All animals had access to water and food ad libitum**.**

### Ethical Approval Statement

All experiments were approved by the Animal Ethical Committee of Walailak University, Thailand **(**WU**-**AICUC**-**64**-**024**)** and were designed by Good Laboratory Practice**.**

### Galactagogue Activity

Six female Wistar virgin rats were used as a sham group**.** Eighteen lactating dams were divided into three groups, each with ten to twelve puppies**.** Group I as a control group, was treated with orally administered distilled water**.** Group II as a positive control was treated with orally administered DOM 2**.**7 mg**/**kg**/**day**.** Group III was treated with orally administered Tri**-**TT 500 mg**/**kg**/**day**.** All lactating rats were treated daily with a gavage syringe from the 3rd to the 14th day of parturition**.** After gavage, milk production was estimated**.** An electronic balance **(**Sartorius Basic Plus**)** was used to measure the milk yield, dam body weight, and weight gain of the pups**.**

The pups were weighed **(**W_1_**)** at 0730 h and isolated from their mother for starved 4 h **27.** At 1130 h, all pups were weighed again **(**W_2_**)** returned to their mother and fed for 1 h**.** At 1230 h, they were weighed**.** The value used was **(**W_2_** -** W_1_**)/**4 for the estimated daily milk yield**.** Weight loss due to metabolic processes in the pup **(**respiration, urination, and defecation**)** during suckling was considered**.** The W_2_ of pup weight was estimated to the daily weight gain of pups **28.** The mean pup milk consumption was calculated as the following formula**:**



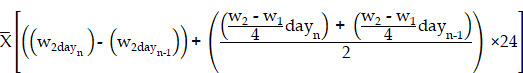



### Blood and Mammary Gland Collections from Lactating Rats

On the 14th day of parturition, lactating rats were weighed and anesthetized by an intraperitoneal dose of 150 mg**/**kg of Thiopental sodium injection** (**Jagsonpal Pharmaceuticals Ltd**.**,**).** Whole blood was collected via the left ventricle **(**cardiac puncture**)** using an 18**-**gauge needle**.** The blood of animals was gradually flowed into the EDTA tubes **(**Ningbo Siny Medical Technology Co**.**, Ltd**)** and incubated at laboratory temperature for 20 minutes**.** The serum was then isolated with a centrifuge machine at 2,000 rpm for 20 minutes, transferred to the Eppendorf tubes, and stored at **-**80°C until use to determine milk production proteins**.** The abdominal inguinal mammary gland chains were carefully dissected, their weights recorded, and the samples were rinsed in saline and fixed in 10**%** formalin for histopathological examination**.**

### Measurement of Milk Production Protein

To evaluate the effect of the Tri**-**TT on milk production, AQP3, AQP5, and LALBA levels in plasma were measured using a commercial enzyme**-**linked immunosorbent assay **(**ELISA**)** kit **(**Sunlong Biotech Co**.**, Ltd**.**, Hangzhou, China**).** AQP3, AQP5, and LALBA levels were examined using the microelisa strip plate that was pre**-**coated with an antibody specific to AQP3 or AQP5, or LALBA**.** Standards or samples were added into wells and combined with the specific antibody**.** Horseradish peroxidase **(**HRP**)** conjugated antibody**-**specific protein was added to each well and incubated**.** After washing, TMB substrate solution was added into the wells to detect the reaction, the color changed to yellow**.** After the addition of the stop solution, the optical density **(**OD**)** was measured spectrophotometrically at an absorbance wavelength of 450 nm by a microplate reader** (**Thermo Scientific**™**Multiskan SkyHigh Microplate Spectrophotometer**).** The mean OD values were derived from the three replicates of the ELISA analysis**.** The sensitivity of AQP3, AQP5, and LALBA ELISA kits was 6 pg**/**ml, 25 pg**/**ml, and 0**.**01 ng**/**ml respectively**.**

### Histopathology of Mammary Glands

Mammary glands tissues were fixed in 10**%** formalin and embedded in paraffin**.** Sections **(**5μm**)** were used for hematoxylin and eosin **(**H&E**)** staining**.** The observation of histologic preparations using a light microscope at 20× magnification**.** Microscopic findings were captured by Olympus CellSens digital imaging software**.** Each sample was photographed separately from 5 random scopes to measure the size of the alveolar diameter**.** Twenty alveoli from each region were then analyzed on average using Free Software Image**-**J** (**NIH, Bethesda, MD, USA**)** in image analysis and μm size unit**.**

### Statistical Analysis

We performed statistical analysis using GraphPad Prism 8 **(**GraphPad Software, San Diego, CA, USA**).** The data are presented as the mean value ± standard error of the mean **(**SEM**).** For the analysis of total milk consumption, protein expression, organ weight and histopathological examination, we used one**-**way analysis of variance **(**ANOVA**).** To analyze the mean pup milk consumption and pups' weight, we conducted a two**-**way ANOVA**.** If significant differences were found, we proceeded with Tukey's HSD significant difference **(**HSD**)** post hoc test**.** Statistical significance was considered at *p < 0**.**05**.**

## Results

### Extract Yield*.*

The result showed that the yield of the aqueous extract of Tri**-**TT was 3**.**71**%.**

### Galactagogue Activity of Tri-TT Remedy in the Lactating Rats

**Figure 1:**
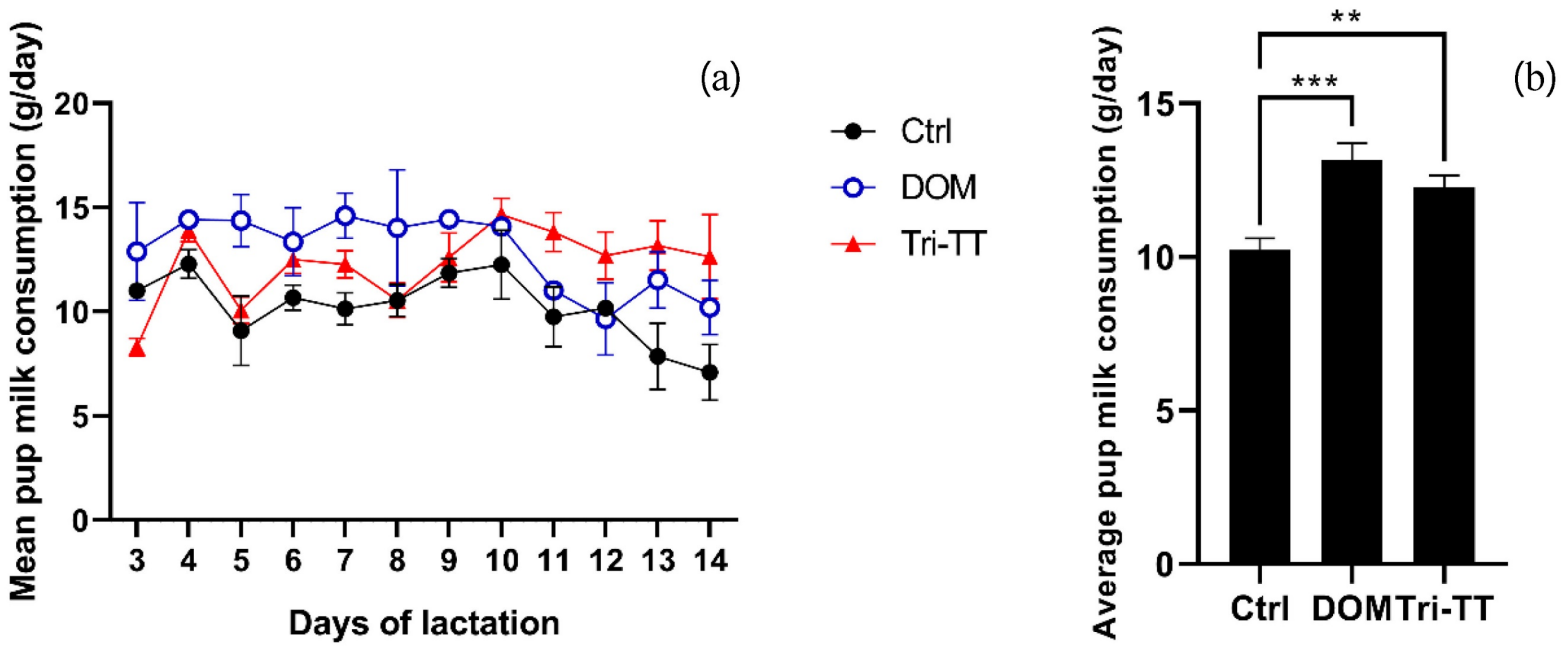
Effect of all extracts on pups' milk consumption (a) mean milk consumption for pups in line graph (b) in bar graph. Statistical significance levels are indicated as: *p < 0.05, **p < 0.01 and ***p < 0.001 compared to the vehicle control, (n=6)

The milk production of lactating rats was assessed by measuring pup's milk consumption, which considers the daily weight gain of fasting pups and the average metabolic loss over 24 hours**.** The results of the study revealed that the mean pup milk consumption in the Tri**-**TT group **(**12**.**27 ± 2**.**26 g**/**day**)** and DOM group **(**13**.**16 ± 3**.**28 g**/**day**)** were significantly higher compared to the control group **(**10**.**23 ± 2**.**32 g**/**day**)** at *p* < 0**.**01 and *p* < 0**.**001, respectively**.** These findings were determined through two**-**way ANOVA analysis **(**Table 2**).** The milk consumption for each day of all extracts is presented in Figure 1**.** Consequently, the mean total milk consumption can be determined by summing the daily milk consumption of all the puppies in the litter**.** In our study, the mean total milk consumption of lactating rats treated with the aqueous extract of Tri**-**TT at 500 mg**/**kg was higher than the control group **(**147**.**21 ± 3**.**12g vs 122**.**76 ± 4**.**05 g**)** throughout the 12**-**day lactation period at *p* < 0**.**01**.** Additionally, lactating rats treated with DOM exhibited the highest total milk production at *p* < 0**.**001**(**Table 2**).**

**Figure 2:**
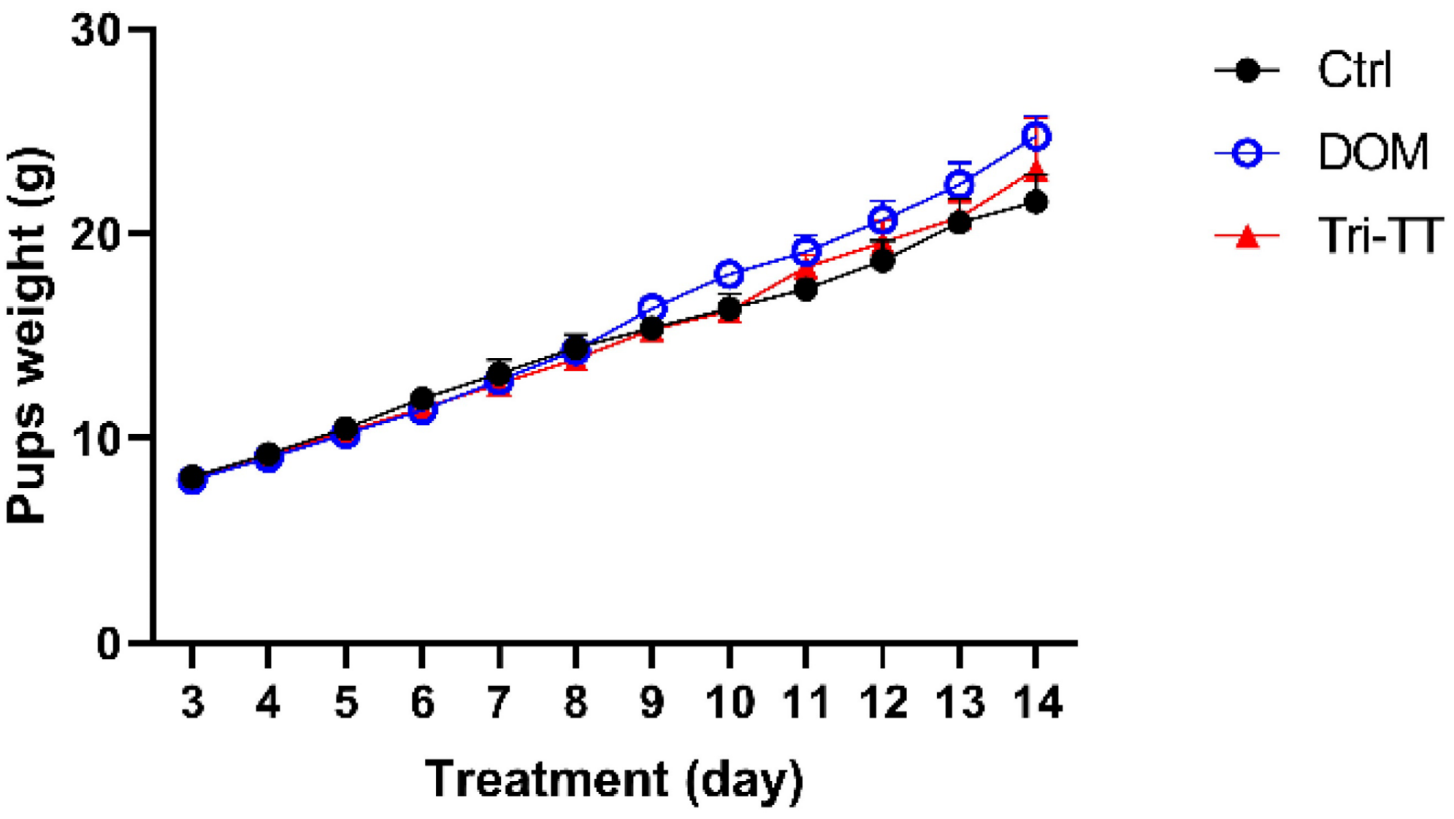
Effect of Tri-TT on pup weight for 12 days, (n=6)

As demonstrated in Figure 2, the daily weight of all pups increases over the period of 12 days of observation**.** The weight gain of each group was calculated from W2 by using the following formula W₂**(**dayₙ**) -** W₂**(**dayₙ**-**₁**)** it will be summed as the total weight gain in each group**.** The initial and final weights and the weight gain of the suckling pups were shown in Table 3**.**

Comparing the treatment groups, the lactating rats treated with DOM displayed a significant difference in the final weight of the pups **(***p* < 0**.**01**)** and a tendency towards significance in the Tri**-**TT group**.** However, when it came to the weight gain of the suckling pups, there was no statistically significant difference observed between the lactating rats treated with the aqueous extract of Tri**-**TT or DOM compared to the control group**.** Analyzing the trends in weight gain for the pups, it was observed that the DOM group exhibited changes starting from the 9th day, while noticeable changes in weight gain for the Tri**-**TT group were observed from the 11th day onwards**.** The control group showed a comparatively lower weight gain trend**.** Overall, these findings indicate that the treatment with Tri**-**TT or DOM had varying effects on the weight gain of the suckling pups, with the DOM group showing more significant differences**.**

### Effect of Tri-TT on body weight and relative organ weight of the lactating rats

**Figure 3:**
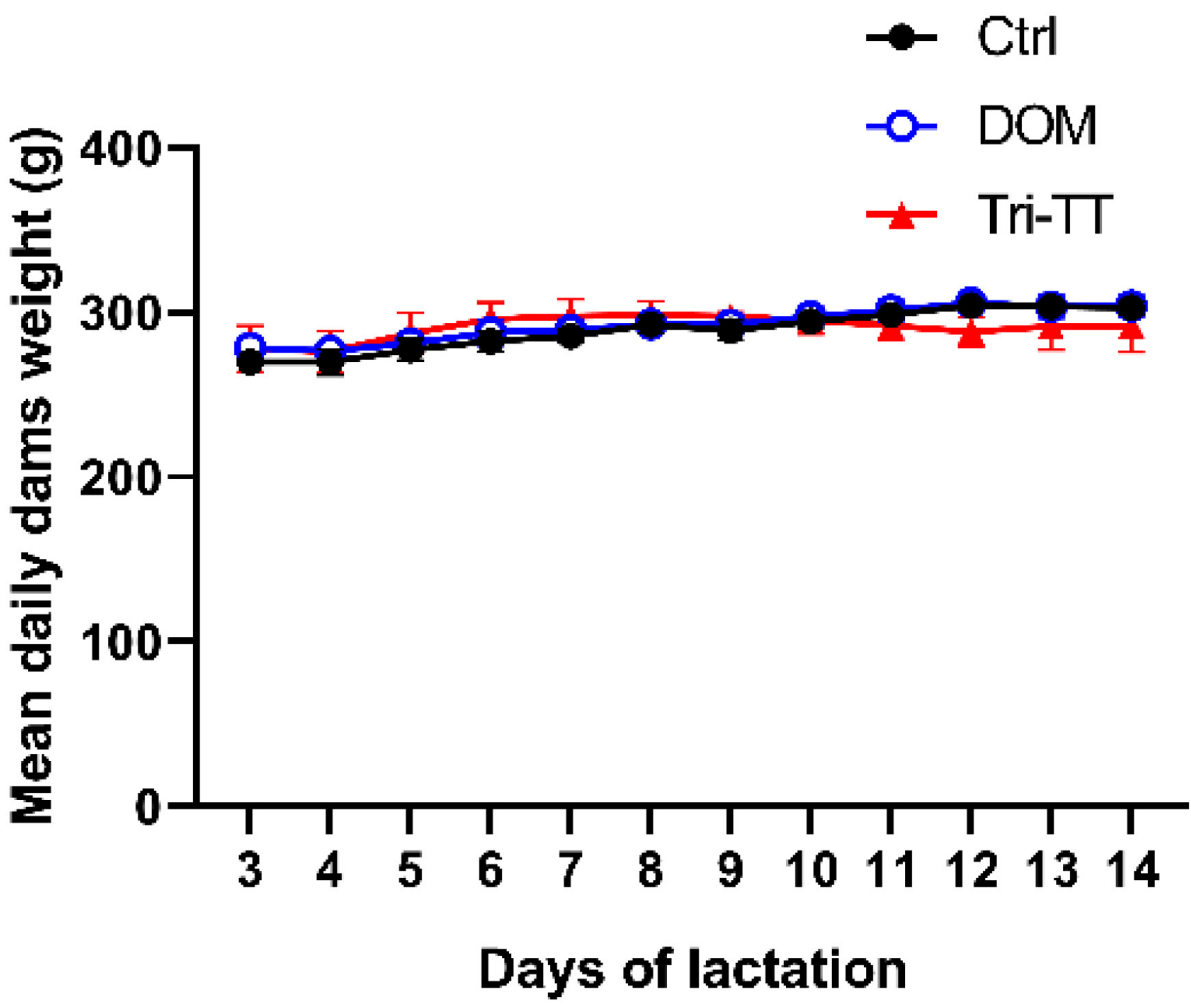
Effect of Tri-TT on daily dams' weight for 12 days, (n=6)

As shown in Figure 3, there was no statistically significant difference in all groups of dam body weight**.** After terminating the lactating rats, the heart, kidney, spleen, lung, liver, mammary gland, and ovary were harvested**.** There were no significant differences related to Tri**-**TT treatment in the relative organ weight of lactating rats** (**Table 4**).**

### The effect of the galactagogue Tri-TT on LALBA, AQP3, and AQP5 proteins

**Figure 4:**
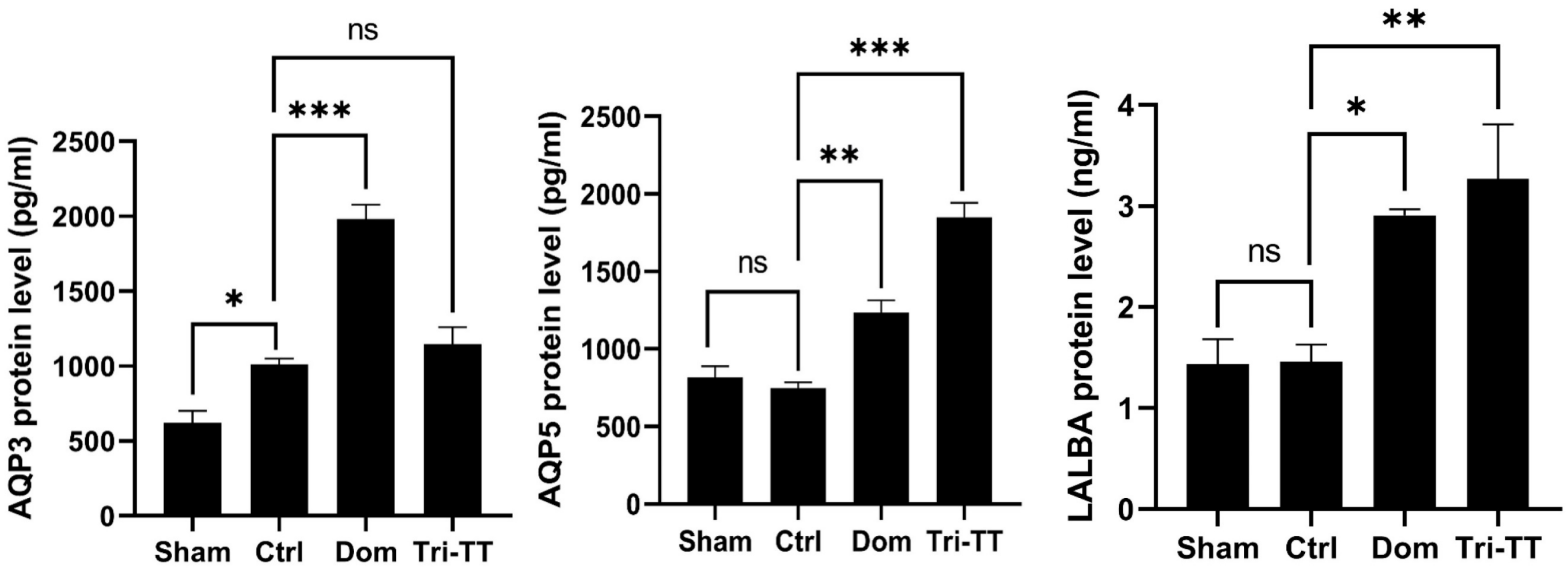
The protein levels of AQP3, AQP5, and LALBA in the lactating rats for 12 days. Data are shown as mean ± SEM. Statistical significance levels are indicated as ns; non-significant, *p < 0.05, **p < 0.01, and ***p < 0.001 compared to the vehicle control, (n=6)

To evaluate the protein, an ELISA kit was performed to assess the protein expression of LALBA, AQP3, and AQP5**.** The results showed the protein levels of AQP5 and LALBA in the lactating rats treated with aqueous extract of Tri**-**TT were significantly increased when compared with the control **(**distilled water**)** group **(**Figure 4**).** In addition, there was no significant difference in the protein levels of AQP3 between the lactating rats treated with aqueous extract of Tri**-**TT and the control groups**.**

### Histopathological examination of the mammary gland

**Figure 5:**
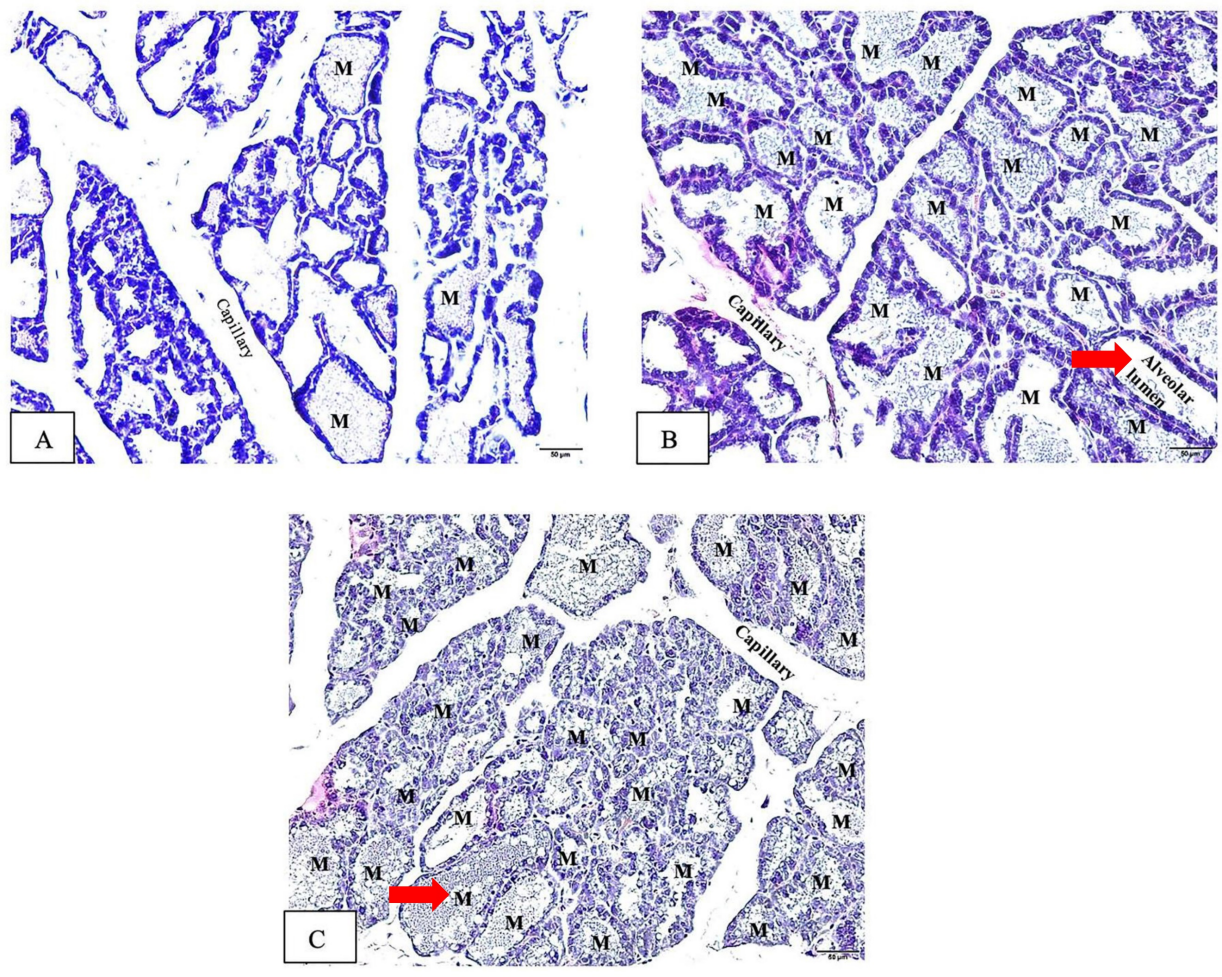
Histopathological examination of the alveolar in the mammary glands induced by Tri-TT at the end of treatment (Hematoxylin Eosin stain, 20x magnification). (A) The negative control (distilled water), (B) The positive control (DOM 2.7 mg/kg), (C) Tri-TT 500 mg/kg. All groups were administered to the lactating rat for 12 days with a sample size of n=6. The letter "M" denotes milk.

In the lactation process, the alveoli undergo changes characterized by an enlargement of the alveolar lumen and increased proliferation of the epithelial cells comprising the alveoli **29, 30.** These alterations reflect the active secretion and synthesis of milk within the mammary gland**.** Our histopathological examination in Figure 5 **(**red arrows**)** illustrates the observed changes at the alveolar level, demonstrating the enlargement of the alveolar lumens in the lactating rats treated with Tri**-**TT **(**Figure 5C**)** and the positive control group **(**Figure 5B**).** These findings suggest that Tri**-**TT may have an impact on the functional aspects of alveolar development and milk production**.**

This result demonstrated that the mean alveolar diameter in the control group was 228**.**93 ± 22**.**25 μm **(**Figure 5A**)** which displays a range in alveolar sizes, notably those that are little and contain little milk**.** Conversely, in the positive control group, 2**.**7 mg**/**kg DOM, the mean was got 350**.**06 ± 42**.**44 μm **(**Figure 5B**).** Each lobule in this group has alveoli that have largely expanded and are filled with breastmilk**.** While the mean in the 500 mg**/**kg Tri**-**TT group was 320**.**03 ± 47**.**31 μm **(**Figure 5C**)**, a slightly larger alveolus was seen in each lobule**.** The size of the mammary gland's alveolar diameter was measured in the Tri**-**TT and DOM groups, and the results showed a significant increase compared to the control group**.** Specifically, in the Tri**-**TT group, the alveolar diameter was increased by 1**.**39 times, while in the DOM group, it was increased by 1**.**53 times**.** These differences were statistically significant with a *p***-**value less than 0**.**05**.** This suggests that both the Tri**-**TT and DOM treatments had a positive effect on increasing the size of the mammary gland's alveolar diameter compared to the control group**.** The Tri**-**TT seems to increase the production of breast milk as seen from the size of the alveolar image, which shows a larger diameter compared to the control group as shown in Figure 5**.**

## Discussion

The purpose of this study was to evaluate the milk production of lactating rats within 12 days of lactation by weighing their litters on a daily weighing as called the weight suckle weight model**.** Several studies have used pup weight and weight gain measurements **4, 5, 27, 31** because litter weight gain per day is estimated to be related to milk production during lactation**.** It was regarded as a rat milk production indicator **31.** The measuring of milk production ended on day 14 because the pups had begun to eat solid food**.**

Our study revealed a significant difference in milk production between the positive control group treated with DOM **(**2**.**7 mg**/**kg**)** and the control group, which aligns with previous studies **4, 7, 32.** Additionally, lactating rats treated with Tri**-**TT at a dose of 500 mg**/**kg exhibited significantly higher milk production compared to the control group**.** Although no significant differences were observed in the weight gain of the suckling pups, the DOM**-**treated group showed a statistically significant difference in final pup weight, which is in line with findings from previous studies **4, 15.** These findings suggest that the aqueous extract of Tri**-**TT or its herbal components may contain chemical constituents that have the potential to stimulate milk production and thereby increase milk quantity**.**

Tri**-**TT has been found to contain various chemical constituents such as quercetin, genistein, and apigenin, which have been associated with enhancing mammary gland development, lactation yield, and the expression of milk production**-**related proteins **25, 33, 34.** Quercetin has been shown to stimulate prolactin secretion **12**, while genistein has an estrogenic influence on prolactin production **25, 33, 34.** Apigenin, another component of Tri**-**TT, has been found to increase the expression of AQP5 in HSG cells, which is important for milk production and secretion **35.** Additionally, previous studies have suggested a significant role of AQP3 in osmoreception in PRL cells and an elevation of LALBA mRNA expression after prolactin stimulation **36.** These findings suggest a potential relationship between prolactin, milk production**-**related proteins, and the action of Tri**-**TT**.** Further experiments would be needed to confirm the specific mechanisms by which Tri**-**TT stimulates milk production, potentially involving the stimulation of prolactin secretion**.** However, the observed increase in milk production and the presence of known milk production**-**related constituents in Tri**-**TT support the hypothesis of its galactagogue effect**.**

Previous studies have investigated the effects of herbal galactagogues on milk production**-**related protein **4, 5.** Liu's study found that a herbal galactagogue mixture increased the expression of AQP3 and AQP5, but not AQP1 **5.** In our study, we focused on AQP**3**, AQP**5**, and LALBA, which are the proteins for milk production**.**

We used DOM as a positive control drug known for its ability to enhance milk production by blocking dopamine activity and promoting PRL production**.** Consistent with other studies **4, 5**, our study showed that administering 2**.**7 mg**/**kg DOM significantly increased the protein levels of AQP3, AQP5, and LALBA, as measured by ELISA**.** Interestingly, when we administered 500 mg**/**kg Tri**-**TT, we also observed a significant increase in the protein levels of AQP5 and LALBA**.** These findings align with previous studies on polyherbal formula **(**PHF**)^4^** and apigenin from herbal extracts **35**, which have shown upregulation of LALBA, AQP1, AQP3, and AQP5**.** Notably, AQP3 was not detected in virgin female rats **37**, and our study also revealed a significant difference between the virgin group and the lactation control group**.** However, it's important to note that our assessment of protein levels was based on serum samples, providing an indirect measurement**.** It is possible that the observed increase in AQP5 and LALBA levels in the serum reflects their synthesis in the mammary gland**.** To further confirm the lactation**-**boosting effect of Tri**-**TT, future studies should evaluate the protein levels of AQP5 and LALBA directly in mammary gland tissue**.**

During pregnancy, the glandular tissue proliferates, with the secretory sections of the gland that develop to form alveoli**.** A bag of contractile myoepithelial cells collects and surrounds the milk**-**secreting alveoli**.** The increase in alveolus diameter is caused by an expansion of the epithelial tissue, which is acting secretory tissue**.** It is interesting that the more there is lactocytes the more the mammary gland will be able to produce milk**.** Our findings showed that the alveolar diameter in the mammary gland increased by 1**.**39 times in lactating rats treated with Tri**-**TT and 1**.**53 times in DOM administration, as well as the PHF expanded alveolar diameter in lactating rats **4.** This demonstrates that the Tri**-**TT dose of 500 mg**/**kg affected the proliferation of myoepithelial cells of the mammary alveolar coat in lactating rats so that the proportion of alveoli grows, leading to the expectation of higher milk production **30.**

Furthermore, Tri**-**TT did not have a safety profile**.** Because this finding was investigated for the first time in lactating rats**.** Lee et al**.'**s finding found that toxicity influences body weight and abnormalities of organs **38** So, we observed the effect of Tri**-**TT on body weight change and abnormalities of organs**.** This result showed no abnormalities in breast tissue or other organs were seen, and there was no significant difference in weight when compared to the control group**.** However, long**-**term safety and the high dose of Tri**-**TT will need to be determined in future studies**.**

## Conclusion

In summary, these findings provide supportive evidence for the galactagogue activity of Tri**-**TT**.** The observed enhancement in milk production that may be associated with Tri**-**TT could potentially be attributed to its ability to widen the alveolar diameter of the mammary gland, thereby facilitating increased milk volume**.** Importantly, the administration of Tri**-**TT did not result in any adverse effects on the body weight or organ weight of the lactating rats**.** This indicates that Tri**-**TT is safe for consumption and does not exhibit acute toxicity**.** Overall, these findings suggest that Tri**-**TT could be a promising natural option for boosting milk production without causing harm to the lactating individual**.** However, further research is needed to elucidate the underlying mechanisms Tri**-**TT promotes lactation and explore its potential applications in supporting breastfeeding mothers**.**

## Figures and Tables

**Table 1:** Phytochemical constituents of Tri-TT and medicinal components.

No.	Scientific name	Plant Part used	Phytoconstituents
1	Tri**-**TT remedy	Roots of* Cassia fistula, Pithecellobium dulce* and* Ficus benjamina*	Picatechin, epigallocatechin, baicalein, genkwanin, (-)-naringenin, pinocembrin, hesperetin, luteolin, apigenin, quercetin 3**′-**O**-**glucuronide and gallic acid **10**
2	*Cassia fistula*	Root	Oxyanthraquinone and phlobaphenes, tannins, and Rhamnetin**-**3**-**O**-**gentiobioside **23, 24**
3	*Pithecellobium dulce*	Root	Quercetin, kaempferol, cyclitol, dulcitol and afezilin, genistein**-**4**'-**O**-**L**-**rhamnopyranoside **25**
4	*Ficus benjamina*	Root	Chlorogenic, *p***-**coumaric, ferulic and syringic acids Gallic acid, Rhein, Anthraquinone, (-)-gallocatechin, Theaflavin**-**3,3'**-**digallate, Flavone **26**

**Table 2:** Milk production of all groups during 12 days of lactation.

Treatment	Mean pup milk consumption (g/day)	Mean total milk consumption during 12 d (g)
Control	10**.**23 ± 2**.**32	122**.**76 ± 4**.**05
Domperidone	13**.**16 ± 3**.**28^***^	157**.**94 ± 2**.**04^***^
Tri**-**TT	12**.**27 ± 2**.**26^**^	147**.**21 ± 3**.**12^**^

The results are presented as mean ± SEM. Statistical significance levels are indicated as: ^*^p < 0.05, ^**^p < 0.01 and ^***^p < 0.001 compared to the control, (n=6)

**Table 3:** Pup's initial, final weight, and weight gain.

Treatment	Mean of initial weight (g)	Mean of final weight (g)	Weight gain (g)
Control	8**.**671 ± 0**.**36	21**.**794 ± 0**.**94	14**.**88 ± 0**.**27
Domperidone	7**.**963 ± 0**.**20^ ns^	24**.**773 ± 0**.**87^ **^	16**.**07 ± 0**.**17^ns^
Tri**-**TT	7**.**786 ± 0**.**09^ ns^	23**.**576 ± 1**.**92^ ns^	15**.**22 ± 0**.**45^ns^

The results were obtained from a sample size of n=6 and are presented as mean ± SEM. Non-statistically significant differences are indicated as ns, while statistically significant differences are denoted as *p < 0.05, **p < 0.01 compared to the control.

**Table 4:** Relative organ weights of lactating rats (percentage of body weight) for 12 days, (n=6)

Parameters	Control	Domperidone	Tri-TT
Heart	0**.**272 ± 0**.**01	0**.**272 ± 0**.**02^ ns^	0**.**267 ± 0**.**01 ^ns^
Kidney	0**.**694 ± 0**.**01	0.658 ± 0.01 ^ ns^	0**.**733 ± 0**.**03 ^ns^
Spleen	0**.**197 ± 0**.**00	0.171 ± 0.01^ ns^	0**.**205 ± 0**.**02 ^ns^
Lung	0**.**343 ± **.**02	0**.**348 ± 0**.**02^ ns^	0**.**355 ± 0**.**01^ ns^
Liver	4**.**642 ± 0**.**11	4**.**486 ± 0**.**10^ ns^	4**.**436 ± 0**.**27^ ns^
Mammary glands	3**.**576 ± 0**.**21	3**.**781 ± 0**.**36^ ns^	4**.**073 ± 0**.**26^ ns^
Ovary	0**.**103 ± 0**.**02	0**.**142 ± 0**.**01^ ns^	0**.**139 ± 0**.**25^ ns^

Report values are mean ± SEM. Non-statistical significance levels are indicated as ns

## Abbreviations

Tri-TT: Tri**-**Than**-**Thip
Ctrl: Control
DOM: Domperidone
AQP3: Aquaporin 3
AQP5: Aquaporin 3
LALBA: Alpha**-**Lactalbumin
